# Serotonin Selectively Increases Detectability of Motion Stimuli in the Electrosensory System

**DOI:** 10.1523/ENEURO.0013-18.2018

**Published:** 2018-05-25

**Authors:** Mariana M. Marquez, Maurice J. Chacron

**Affiliations:** 1 Department of Physiology, McGill University, Montreal H3G1Y6, Canada

**Keywords:** burst firing, feedback, neuromodulation, serotonin, weakly electric fish

## Abstract

Serotonergic innervation of sensory areas is found ubiquitously across the central nervous system of vertebrates. Here, we used a system’s level approach to investigate the role of serotonin on processing motion stimuli in the electrosensory system of the weakly electric fish *Apteronotus albifrons*. We found that exogenous serotonin application increased the firing activity of pyramidal neural responses to both looming and receding motion. Separating spikes belonging to bursts from those that were isolated revealed that this effect was primarily due to increased burst firing. Moreover, when investigating whether firing activity during stimulation could be discriminated from baseline (i.e., in the absence of stimulation), we found that serotonin increased stimulus discriminability only for some stimuli. This is because increased burst firing was most prominent for these. Further, the effects of serotonin were highly heterogeneous, with some neurons displaying large while others instead displaying minimal changes in responsiveness following serotonin application. Further analysis revealed that serotonin application had the greatest effect on neurons with low baseline firing rates and little to no effect on neurons with high baseline firing rates. Finally, the effects of serotonin on sensory neuron responses were largely independent of object velocity. Our results therefore reveal a novel function for the serotonergic system in selectively enhancing discriminability for motion stimuli.

## Significance Statement

Previous studies have suggested that the role of serotonin in the electrosensory system was to enhance perception of and neural responses to stimuli associated with same-sex conspecifics. However, these have focused on stationary stimuli. Here, we focused on motion stimuli that are typically associated with a different behavioral context (e.g., prey capture). Although exogenous serotonin application increased burst firing to both looming and receding motion, detectability was significantly enhanced only for receding motion that gave rise to a large excitatory response. We propose that serotonin selectively enhances neural responses to stimuli that elicit electrosensory feedback.

## Introduction

Understanding how sensory input is processed by the brain to give rise to behavioral responses remains a central problem in systems neuroscience. Such understanding is complicated by the fact that sensory systems must constantly adapt to natural stimuli whose statistics vary in time (Wark et al., 2007; Sharpee et al., 2014). Such adaptation is thought to be achieved in part by neuromodulators such as serotonin (Hurley et al., 2004; Berger et al., 2009; Marder, 2012). Centrifugal serotonergic fibers emanate from the Raphe nuclei and innervate multiple sensory brain areas. Although evolutionary studies have shown remarkable conservation of this system across vertebrate species (Parent, 1981), previous studies have shown that serotonergic fibers make diverse connection patterns (Foehring et al., 2002; Thompson and Hurley, 2004), thereby causing a wide range of effects on neural activity (Waterhouse et al., 1990; Hurley and Pollak, 2001, 2005; Petzold et al., 2009; Hurley and Sullivan, 2012), and thereby suggesting that the functional role of serotonin is to selectively enhance/suppress neural responses to stimuli associated with different behavioral context(s). Significant insight as to the functional role of serotonergic pathways is likely to be gained by studying sensory systems with well-characterized neural circuits whose responses to natural stimuli associated with different behavioral contexts are well-understood.

The electrosensory system of weakly electric fish provides a convenient model system to study neuromodulation in vertebrate sensory systems because of well-characterized anatomy and physiology, as well as well-described natural stimuli that can easily be reproduced in the laboratory and will give rise to appropriate behavioral responses (Berman and Maler, 1999; Chacron et al., 2011; Márquez et al., 2013; Clarke et al., 2015a). These fish generate a quasi-sinusoidal signal called the electric organ discharge (EOD) around their body, which allows them to explore the environment and communicate with conspecifics. Peripheral electrosensory afferents detect changes in EOD amplitude and relay this information to pyramidal cells within the electrosensory lateral line lobe (ELL). Pyramidal cells also receive large amounts of feedback including neuromodulatory input (Sas and Maler, 1983, 1987; Johnston et al., 1990; Deemyad et al., 2011; Toscano-Márquez et al., 2013) whose functions comprise gain control (Bastian, 1986b), adaptive cancellation of redundant stimuli (Bastian, 1999; Bastian et al., 2004; Bol et al., 2011), and selective enhancement of neural responses (Ellis et al., 2007a; Deemyad et al., 2013). In particular, recent studies have focused on understanding the role of serotonergic projections onto ELL pyramidal cells: it was found that such input increases ELL pyramidal cell responsivity to stimuli associated with same-sex conspecifics (Deemyad et al., 2013) by increasing excitability through inhibition of potassium currents (Deemyad et al., 2011). Interestingly, another study has used *in vivo* voltammetry to measure serotonin levels in ELL: it was found that levels increased following stimulation associated with conspecifics (Fotowat et al., 2016). These studies suggest that the primary role of the serotonergic system is to facilitate neural processing of stimuli associated with social interactions. However, weakly electric fish must also electrolocate (e.g., find relevant objects such as prey) in their environment, which is associated with movement (Nelson and MacIver, 1999). While ELL pyramidal cell responses to lateral (Bastian, 1981; Chacron et al., 2009; Khosravi-Hashemi and Chacron, 2014) as well looming and receding motion (Clarke et al., 2014, 2015b; Clarke and Maler, 2017) have been studied, the effects of neuromodulatory input on these has not been investigated to date.

Here, we investigated the effects of serotonergic input on the responses of ELL pyramidal cells in response to looming and receding motion. Previous studies have extensively investigated ELL pyramidal cell responses to such motion (Clarke et al., 2014, 2015b; Clarke and Maler, 2017). In particular, it was found that objects that inhibit pyramidal cell activity during the looming phase of motion will cause a large burst of spikes during the receding phase (Clarke et al., 2014, 2015b). This receding response occurs even when the object is stationary for a few seconds and is generated by descending pathways while the response to looming motion is instead generated by feedforward pathways (Clarke and Maler, 2017). We found that exogenous serotonin application increased the firing activity of pyramidal cells during both the looming and receding phases of motion, which was due to increased burst firing. However, serotonin enhanced discriminability for receding motion only when this stimulus elicited a prominent excitatory response mainly generated by feedback inputs, independently of the object’s velocity. Our results thus provide the first experimental evidence that the serotonergic system is involved in increasing discriminability of neural responses to receding but not looming motion.

## Materials and Methods

### Animals and surgery

Specimens of the weakly electric fish *Apteronotus albifrons* were acquired from tropical fish suppliers and acclimated to laboratory conditions according to published guidelines (Hitschfeld et al., 2009). A total of 27 animals of either sex were used in these experiments. All animal procedures were performed in accordance with the institutional animal care committee’s regulations.

Surgical procedures have been described in detail elsewhere (Martinez et al., 2016; Hofmann and Chacron, 2017). Briefly, the fish was paralyzed by intramuscular injection of tubocurarine (1 µg/g; Sigma-Aldrich), placed in the recording tank and respirated with oxygenated water flowing at a constant rate of ∼10 ml/min. A portion of the animal’s head was kept out of water and anesthetized with topical application of lidocaine ointment (2%, Western Medical Supply). A small incision was made over the hindbrain and a metal post was glued to the most anterior section of the exposed skull to stabilize the animal’s position in space. A small craniotomy was then made to access the ELL.

### Electrophysiology

The brain anatomy of *A. albifrons* is very similar to that of *Apteronotus leptorhynchus* (Maler, 1979, 1981; Maler et al., 1991). We recorded extracellularly record from ELL pyramidal cells (*n* = 27) using techniques similar to those used previously (Martinez et al., 2016). Based on recording depth as well as electrode placement relative to surface landmarks (e.g., To vein and its afferents; Krahe et al., 2008), it is likely that our recordings were from the lateral segment, although it is possible that some recordings were from the adjacent centro-lateral segment. Previous studies performed in *A. leptorhynchus* have shown that the lateral segment displayed the greatest density of serotonergic innervation (Deemyad et al., 2011). Recordings were made using electrodes filled with Woods Metal and plated with both gold and platinum (Frank and Becker, 1964). The electrodes tip diameter was typically ∼5 µm. All recordings were amplified (A-M Systems 1700), digitized at a 10-kHz sampling rate (CED 1401; Spike2 version 8.1 software; Cambridge Electronic Design), and stored for subsequent analysis.

### Pharmacology

Glutamate (3 mM; Sigma-Aldrich) and serotonin (1 mM; Sigma-Aldrich) were dissolved in saline (111 mM NaCl, 2 mM KCl, 2 mM CaCl_2_, 1 mM MgSO_4_, 1 mM NaHCO_3_, and 0.5 mM NaH_2_PO_4_; Sigma-Aldrich) for application. Drug application electrodes were two-barrel KG-33 glass micropipettes (OD = 1.5 mm, ID = 0.86 mm, A-M Systems) pulled by a vertical micropipette puller (Stoelting) and subsequently broken to attain a final tip diameter of ∼10 µm. During recordings we used a picospritzer (Parker Hannifin) for separate delivering small puffs of glutamate or serotonin. We first used excitatory responses to glutamate to verify that we were in the vicinity of the cell being recorded from, as evidenced from a short latency (<2 ms) increase in spiking activity following drug application. Drugs were typically delivered at 15–25 psi during 150 ms, as done previously (Toporikova and Chacron, 2009; Deemyad et al., 2013; Huang et al., 2016). We note that previous studies have repeatedly shown that application of saline alone in this manner does not significantly alter ELL pyramidal cell activity (Bastian, 1993; Toporikova and Chacron, 2009; Deemyad et al., 2013; Huang et al., 2016).

### Stimulation

#### Moving object

*A. albifrons* has a neurogenic electric organ which discharge (EOD) is not affected by immobilization with tubocurarine (Hitschfeld et al., 2009). As a consequence, the immobilized fish is still able to sense local perturbations in its EOD amplitude caused by objects with different conductivity than the surrounded water, such as plastic or metal. The stimulus consisted of a plastic or metal sphere (1.5 cm in diameter) controlled by a pen plotter (HP 7035B) and located at a given cell’s receptive field (RF) center (see section <On and off type cell classification>). The object’s trajectory was a sequence of looming motion toward the fish over a distance of 6 cm followed by a 2-s pause at 0.5 cm away from the skin surface and receding motion over the same distance followed by a 2-s pause at 6.5 cm away from the animal. The stimulation protocol consisted of 50 repetitions or trials played at four different velocities: 3, 6, 8, and 12 cm/s. These values were chosen to match the behaviorally relevant range observed during locomotion studies (Bastian, 1982; Rose and Canfield, 1993a,b; Nelson and MacIver, 1999; Cowan and Fortune, 2007).

#### ON and OFF type cell classification

Within the ELL, there are two types of pyramidal cells that can be classified based on their responses to stimuli (Bastian, 1981; Martinez et al., 2016): ON cells respond preferentially to increases in EOD amplitude while OFF cells respond preferentially to decreases in EOD amplitude. We used the same methodology as Martinez et al. (2016) to classify each pyramidal cell recorded from. Specifically, a noisy amplitude modulation stimulus (0–120 Hz) was played via two electrodes located 15 cm on each side of the animal. The spike-triggered average (STA) is the mean stimulus waveform that triggers an action potential and was obtained by averaging the stimulus waveforms within a 50-ms time window surrounding each spike:

$$STA\left(t\right)=\frac{1}{N}\sum_{i=1}^{n}S(t-t_{i})$$

where *S(t)* is the time-varying noisy AM stimulus. The cell was classified as being ON-type if the slope of the STA within a time window of 10 ms centered at 7 ms was positive and classified as being OFF-type if the slope of the STA was negative. We note that the negative offset of 7 ms was used to account for the axonal transmission delay from the skin surface to the ELL (Chacron et al., 2003). RFs from ELL pyramidal cells were located using a local stimulus dipole and a 4-Hz sinusoidal amplitude modulation as done previously (Bastian et al., 2002; Chacron et al., 2009). Previous studies have shown that receding motion triggers paradoxical responses to electrosensory contrast (i.e., ON-type cells respond preferentially to receding motion of a negative contrast object while OFF-type cells respond preferentially to receding motion of a positive contrast object; Clarke et al., 2014). Recordings were made when stimulating with either of a matching or non-matching contrast paradigm. For the matching contrast paradigm (*n* = 9), we used a metal sphere when recoding from ON cells and a plastic sphere when recording from OFF cells as done previously (Clarke et al., 2015b; Clarke and Maler, 2017). For the non-matching contrast paradigm (*n* = 18), we used a metal sphere when recoding from OFF cells and a plastic sphere when recording from ON cells as done previously (Clarke et al., 2015b; Clarke and Maler, 2017).

### Data analysis

All analysis was performed offline using Spike2 and custom written scripts in MATLAB (MathWorks). Action potential times were defined as the times for which the signal crossed a suitably chosen threshold.

#### Spontaneous activity

The spontaneous firing rate of every neuron was calculated from 100 s of activity before any stimulus was presented. All quantities are reported as mean ± SE.

#### Distinguishing bursts from isolated spikes

**Figure 1.**
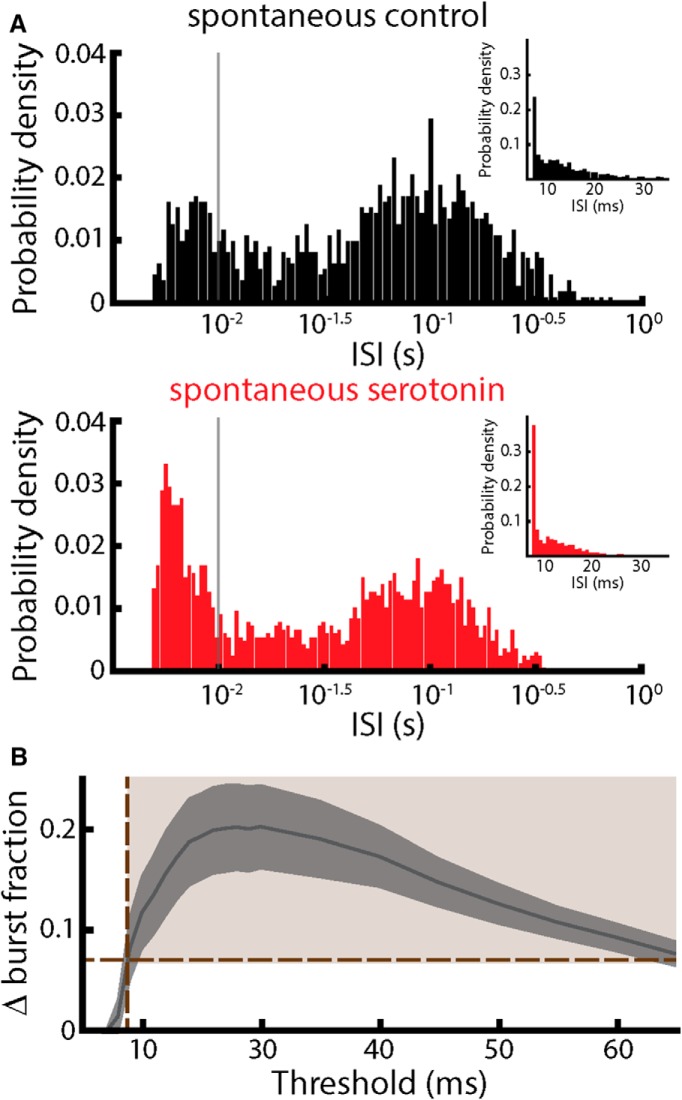
Effects of serotonin on spontaneous ELL pyramidal cell activity. ***A***, Top, Interspike interval (ISI) histogram from a typical ELL pyramidal cell before serotonin application with the *x*-axis plotted on a logarithmic scale. Inset, ISI histogram from the same cell but with the *x*-axis plotted on a linear scale. Bottom, ISI histogram from the cell after serotonin application with the *x*-axis plotted on a logarithmic scale. Inset, ISI histogram from the same cell after serotonin application but with the *x*-axis plotted on a linear scale. Note that a clear bimodality can be seen from the main panels. The vertical gray lines indicate the chosen burst threshold of 10 ms. ***B***, Change in burst fraction due to serotonin application averaged over our dataset as a function of the burst threshold. The solid line shows the population average and the gray band shows ±1 SEM.

We used an algorithm to distinguish between spikes that belong to bursts (i.e., burst spikes) and those that do not (i.e., isolated spikes). Specifically, spikes are part of bursts if they are separated by a time interval that is less than the threshold and are considered isolated spikes otherwise. This algorithm has been used extensively before in the electrosensory system (Oswald et al., 2004; Avila-Akerberg et al., 2010; Khosravi-Hashemi et al., 2011; Khosravi-Hashemi and Chacron, 2012, 2014). We chose a threshold of 10 ms in accordance with these previous studies. Furthermore, this value corresponded to a well-defined mode of the interspike interval distribution under spontaneous activity for our dataset (Fig. 1). Burst fraction was computed as the ratio of the number of spikes that belong to bursts to the total number of spikes.

#### Response to moving objects

Responses to moving objects were accumulated as sequences of time where action potentials occurred (spike times) and were converted to binary sequences by discretizing time into bins of 0.1 ms width. Peristimulus time histograms (PSTHs) were generated by building a histogram from the binary sequences of spike times, diving the values by the bin size, and then multiplying the result by the number of trials and smoothing with a 200-ms-long box car filter. We used receiver operating characteristic (ROC) analysis (Green and Swets, 1966) to quantify the ability of an ideal observer to distinguish between neural responses to either looming or receding motion stimuli and those obtained when the object was stationary and far away (6.5 cm) from the animal (i.e., baseline). Specifically, spike count distributions were obtained from neural responses over the course of the entire looming and receding phases of motion and compared to that obtained from baseline over the same time period. The probabilities of correct detection [P(correct)] and false alarm [P(false alarm)] were computing by integrating the spike count distributions up to a variable threshold. The ROC curve was then obtained by plotting P(correct) as a function of P(false alarm) while systematically varying the threshold. Detectability was quantified by computing the as the area under the ROC curve (auROC). A value of 1 for auROC indicates perfect discrimination while a value of 0.5 indicates chance level.

#### Statistics

Statistical significance was assessed through a Student’s *t* test or a Wilcoxon signed-rank test for paired measurements at the *p* = 0.05 level. For multiple comparisons, statistical significance was assessed through one-way ANOVA at the *p* = 0.05 level. Correlations were calculated using a Pearson correlation test or Spearman correlation test.

## Results

### Serotonin application increases pyramidal cell excitability and burst firing

**Figure 2.**
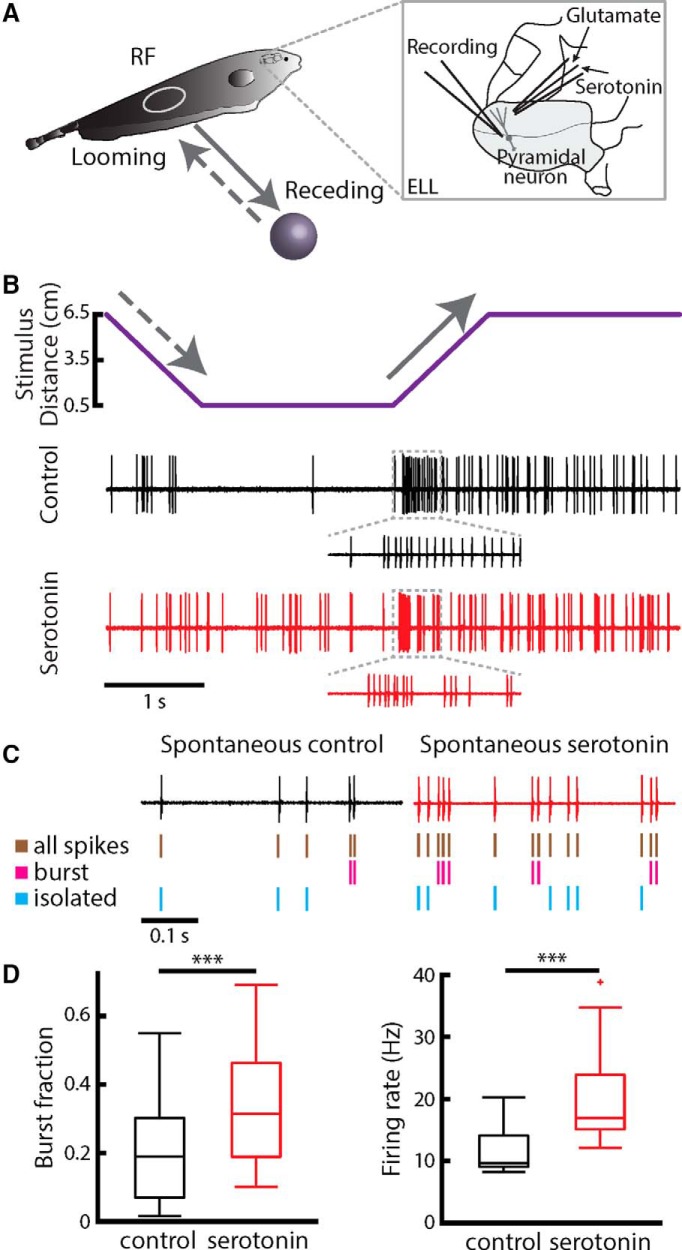
Serotonin affects ELL pyramidal cell responses to moving objects through burst firing. ***A***, Schematic representation of the experimental setup. The stimulus (plastic or metal sphere) is aligned to the RF of a given cell and follows a looming (dashed gray line) or a receding (solid gray line) trajectory while the cell’s response is recorded extracellularly. The inset shows the recording electrode that is placed near the cell and a double barrel pipette with glutamate and serotonin that is placed near the cell’s apical dendritic tree. ***B***, Schematics showing one full stimulus cycle and the response of a representative neuron. In a non-matching contrast paradigm, during control condition (black trace) the neuron responds preferentially to receding motion with a group of action potentials occurring at a high frequency rate of discharge (i.e., a burst). This response is enhanced after serotonin application (red trace, compare insets). ***C***, Spiking responses under control (black) and after (red) serotonin application. Shown are the full spike trains (brown), burst spikes (magenta), and isolated spikes (cyan). ***D***, Population-averaged burst fraction (i.e., the fraction of spikes that belong to bursts; left) and mean firing rate (right) before (black) and after (red) serotonin application (*n* = 13). Stars indicate statistical significance using a signed-rank test (*p* = 0.0002) and a paired *t* test (*t*_(12)_ = −3.4530, *p* = 0.005), respectively.

We recorded extracellularly from ELL LS pyramidal neurons (*n* = 27) in *A. albifrons* and measured their responses to moving objects in awake behaving animals before and after exogenous serotonin application (Fig. 2*A*). Focal serotonin application was achieved by inserting a double-barrel electrode in the ELL molecular layer with one barrel containing glutamate and the other serotonin that was connected to a picrospritzer (Fig. 2*A*, right). Glutamate ejection was used to ascertain that the double barrel electrode was located near the apical dendritic tree of the pyramidal cell being recorded from. Previous studies performed in *A. leptorhynchus* have shown that, when using a non-matching contrast paradigm (i.e., stimulating ON cells with a plastic object and OFF cells with a metal object), ELL pyramidal neurons respond to receding motion by increasing their firing rate and more specifically, by firing more burst spikes (Clarke et al., 2014). Our results show that ELL pyramidal cells in *A. albifrons* respond to receding motion in a similar manner (Fig. 2*B*, top). Consistent with previous results obtained in *A. leptorhynchus* (Deemyad et al., 2013), we found that serotonin application increased pyramidal cell excitability for spontaneous activity (i.e., in the absence of stimulation but in the presence of the animal’s unmodulated EOD; Fig. 2*C*). Specifically, pyramidal cells displayed an increased tendency to fire packets of action potentials followed by quiescence (i.e., bursts), as revealed by separating the spike train into burst (Fig. 2*C*, magenta) and isolated spikes (Fig. 2*C*, cyan) using an interspike interval threshold criterion. Overall, serotonin application increased the cell’s tendency to fire bursts, as quantified by a large increase in burst fraction (i.e., the fraction of spikes that belong to bursts; Fig. 2*D*, left), which led to an increase in the overall firing rate (Fig. 2*D*, right).

### Serotonin enhances the firing rate through increased burst firing during all phases of stimulation

**Figure 3.**
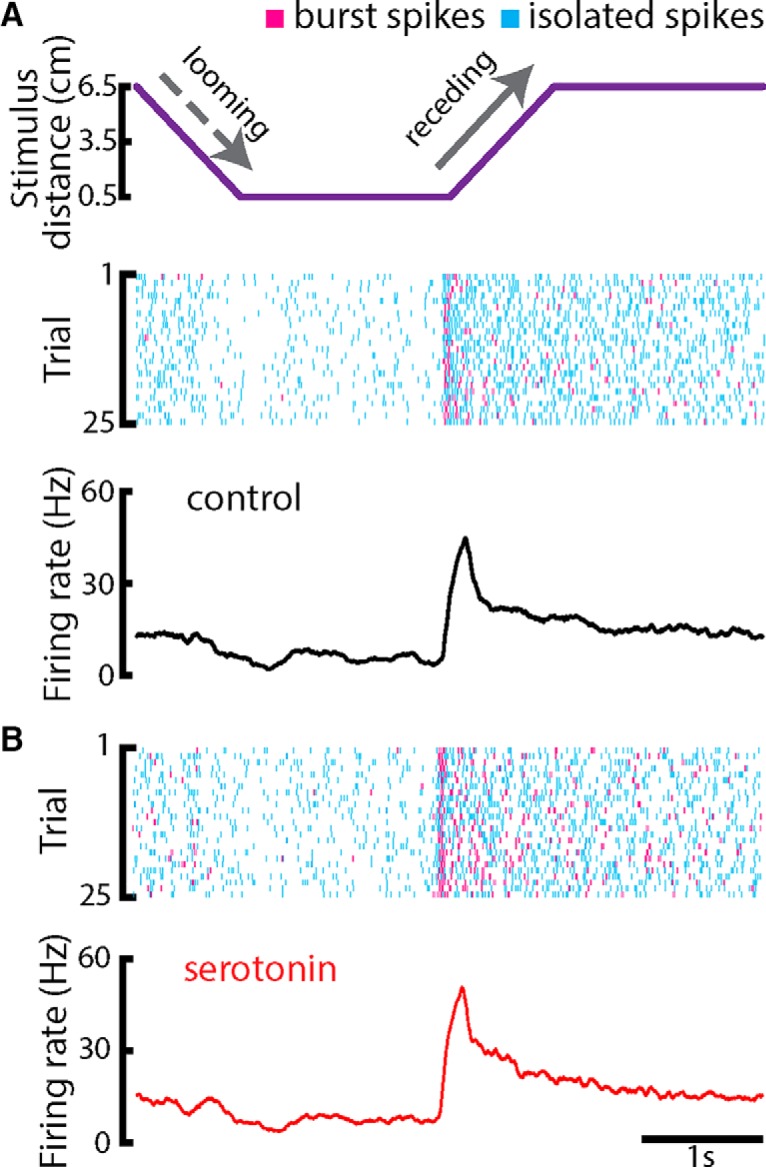
Serotonin promotes burst firing in response to motion stimuli during a non-matching contrast paradigm. ***A***, Top, object position (lateral distance to the animal’s skin surface) as a function of time. The object’s speed during both looming and receding motion was 8 cm/s. Middle, Raster plot showing an example ELL pyramidal neuron’s spiking response to 25 stimulus presentations (i.e., trials) during control condition. The spikes that belong to bursts are shown in magenta, whereas isolated spikes are shown in cyan. Bottom, PSTH of the neuron computed from 50 trials using all spikes. ***B***, Top, raster plot showing the example neuron’s response to 25 trials after serotonin application. Bottom, PSTH computed from 50 trials using all spikes.

We next investigated the effects of serotonin on responses to looming and receding motion. While looming motion causes decreased spiking activity (Fig. 3*A*,*B*, dashed gray arrow), receding motion instead causes increased spiking activity (Fig. 3*A*,*B*, solid gray arrow). Overall, serotonin application gave rise to increased burst firing during all phases of stimulation (Fig. 3*A*,*B*). While there was some variability in responses to repeated stimulus presentations (Fig. 3*A*,*B*), responses showed strong similarity and were thus averaged to the PSTH. The PSTH response of a typical ELL pyramidal cell before and after serotonin application are shown in the bottom panel of Figure 3*A*. Overall, looming motion led to a decrease in firing rate which somewhat recovered while the object remains stationary and close to the animal (Fig. 3*A*, dashed gray arrow). There was a sharp increase in firing rate following the onset of receding motion (Fig. 3*A*, solid gray arrow) that then slowly adapted back toward the baseline value while the object remained stationary and far away from the animal.

**Figure 4.**
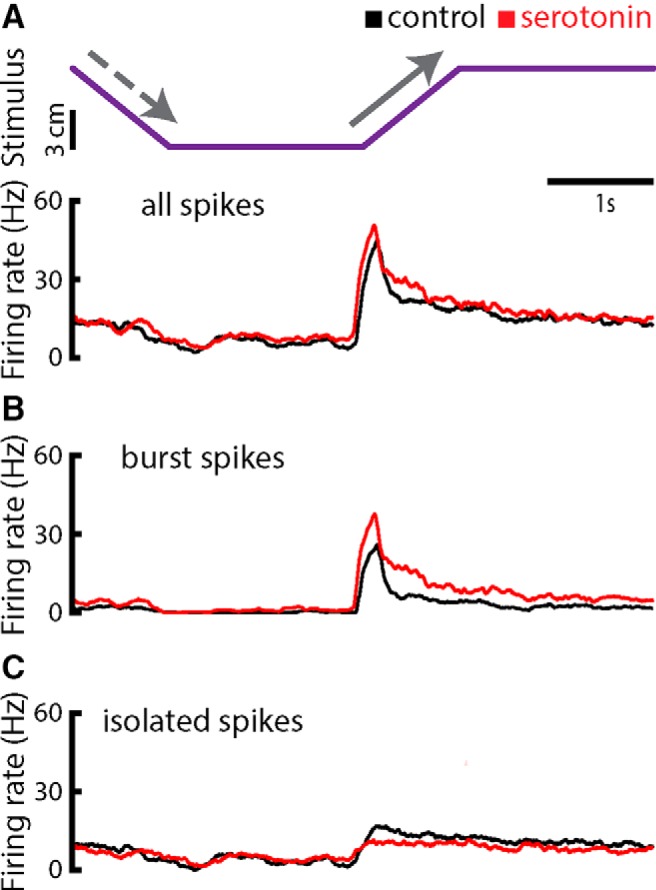
Serotonin increases the firing rate during all phases of stimulation. ***A***, Top, object position (lateral distance to the animal’s skin surface) as a function of time. The object’s speed during both looming and receding motion was 8 cm/s. Bottom, PSTH from an example ELL pyramidal neuron computed from all spikes before (black) and after (red) serotonin application. There was a decrease in firing rate during the looming phase (dashed arrow). The firing rate reached its maximum value right after the onset of receding motion during both control (black trace) and after serotonin application (red trace). Note that the firing rate was overall higher during all stimulus phases after serotonin application. ***B***, PSTH from the example ELL pyramidal neuron computed from burst spikes before (black) and after (red) serotonin application. ***C***, PSTH from the example neuron computed using isolated spikes before (black) and after (red) serotonin application.

**Figure 5.**
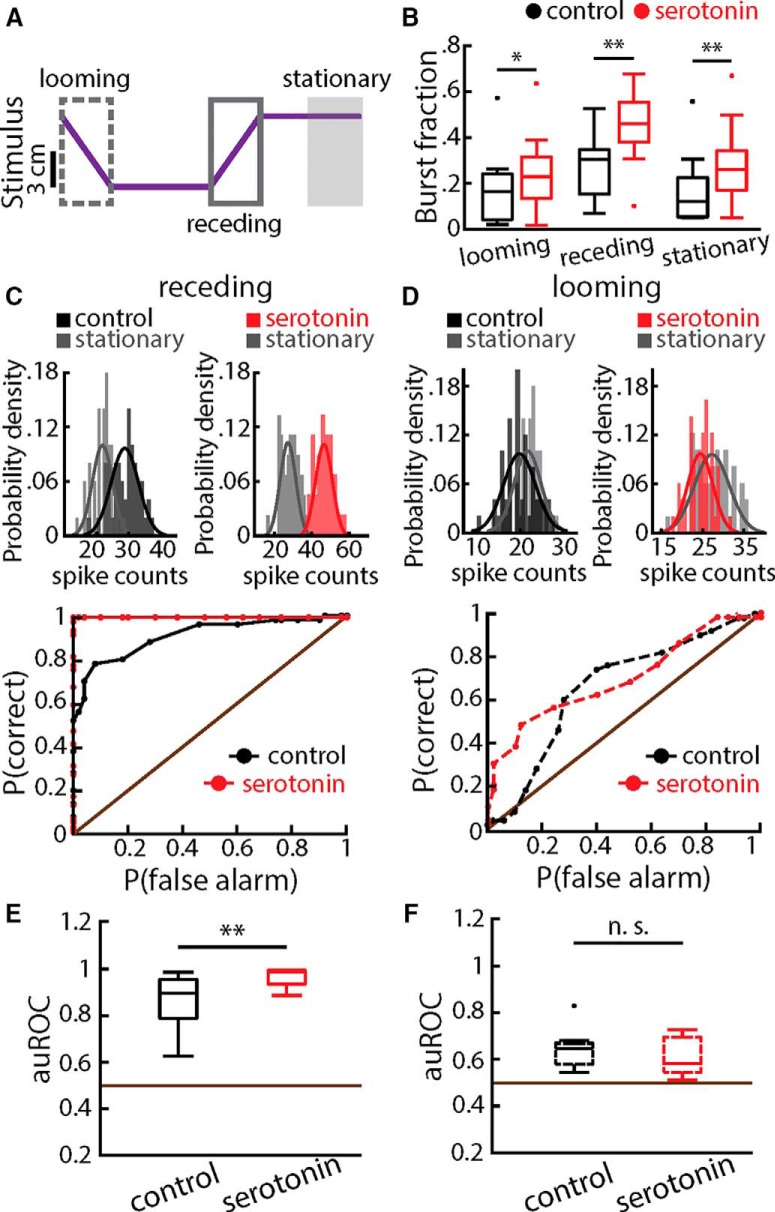
Serotonin increases stimulus detectability for receding but not for looming motion. ***A***, Object position (lateral distance to the animal’s skin surface) as a function of time. The neural responses during looming (dashed gray box) and receding (middle solid gray box) gray boxes were compared to the baseline activity while the object was stationary and located far away from the animal (right gray box). ***B***, Population-averaged burst fractions before (black) and after (red) serotonin application during looming (left), receding (middle), and baseline (right). Stars indicate statistical significance using a paired *t* test (looming: *t*_(12)_ = −2.70, *p* = 0.03, *n* = 13; receding: *t*_(12)_ = −3.35, *p* = 0.009, *n* = 13; baseline: *t*_(12)_ = −3.08, *p* = 0.01, *n* = 13). ***C***, Top, spike count distributions obtained during baseline (gray) and during receding stimulation (black or red) before (left) and after (right) serotonin application for an example ELL pyramidal cell. Best-fit Gaussian curves are superimposed on each distribution. Bottom, ROC curves from this same example neuron before (black) and after (red) serotonin application. ***D***, Same as ***C*** but for looming motion. ***E***, Population-averaged values for the auROC before (black) and after (red) serotonin application for receding motion. The horizontal brown line indicates the chance level. A significant increase was observed after serotonin application (signed-rank test, *p* = 0.0007, *n* = 13). ***F***, Same as ***E*** but for looming motion. No significant change was observed (*t* test, *t*_(12)_ = 1.08, *p* = 0.301, *n* = 13).

Serotonin application led to an overall increase in the average firing rate of ELL pyramidal cell during all phases of stimulation (Fig. 4*A*, bottom panel). Since our results have shown that the effect of serotonin was to increase burst firing during baseline conditions, we hypothesized that increased responses were due to increases in the tendency to fire bursts during receding motion. To test this hypothesis, we only considered spikes that were part of bursts (i.e., burst spikes) or not (i.e., isolated spikes) to compute the PSTH. Our results confirmed our hypothesis in that the PSTHs obtained from burst spikes but not isolated spikes showed a large increase in firing during the receding phase of motion (Fig. 4*B*,*C*). These results were seen across our dataset, as serotonin increased the burst fraction during both looming and receding motion, as well as when the object was stationary and far away from the animal (i.e., “baseline”; Fig. 5*A*,*B*). However, the increase in burst fraction during looming motion and during baseline were similar, while the increase during receding motion was more substantial (Fig. 5*B*).

### Serotonin application increases the detectability of receding but not looming motion when using a non-matching contrast paradigm

It is important to note that, to be detected, sensory input must perturb the ongoing activity of ELL pyramidal cells in the absence of stimulation. Thus, we next used ROC analysis to test whether serotonin affected the discriminability of looming and receding motion. Specifically, spike count distributions obtained during looming and receding stimulation were compared to that obtained during the baseline (Fig. 5*A*). The upper panels of Figure 5*C* show the spike count distributions from an example cell before (upper left) and after (upper right) serotonin application. It is seen that the distributions are more separable after serotonin application, which is reflected in the ROC curve (Fig. 5*C*, middle panel). Overall, serotonin application significantly increased receding motion stimulus detectability as quantified by computing the auROC (Fig. 5*C*, bottom panel). For comparison, spike count distributions obtained during looming motion are shown in the upper panel of Figure 5*D*. It is seen that serotonin application did not change the discriminability between the spike count distributions (Fig. 5*D*, compare left and right upper panels), which is reflected in the ROC curves (Fig. 5*D*, middle panel). Overall, serotonin application did not affect looming motion stimulus detectability as the auROC values obtained before and after serotonin application were not significantly different from one another (Fig. 5*D*, bottom panel).

**Figure 6.**
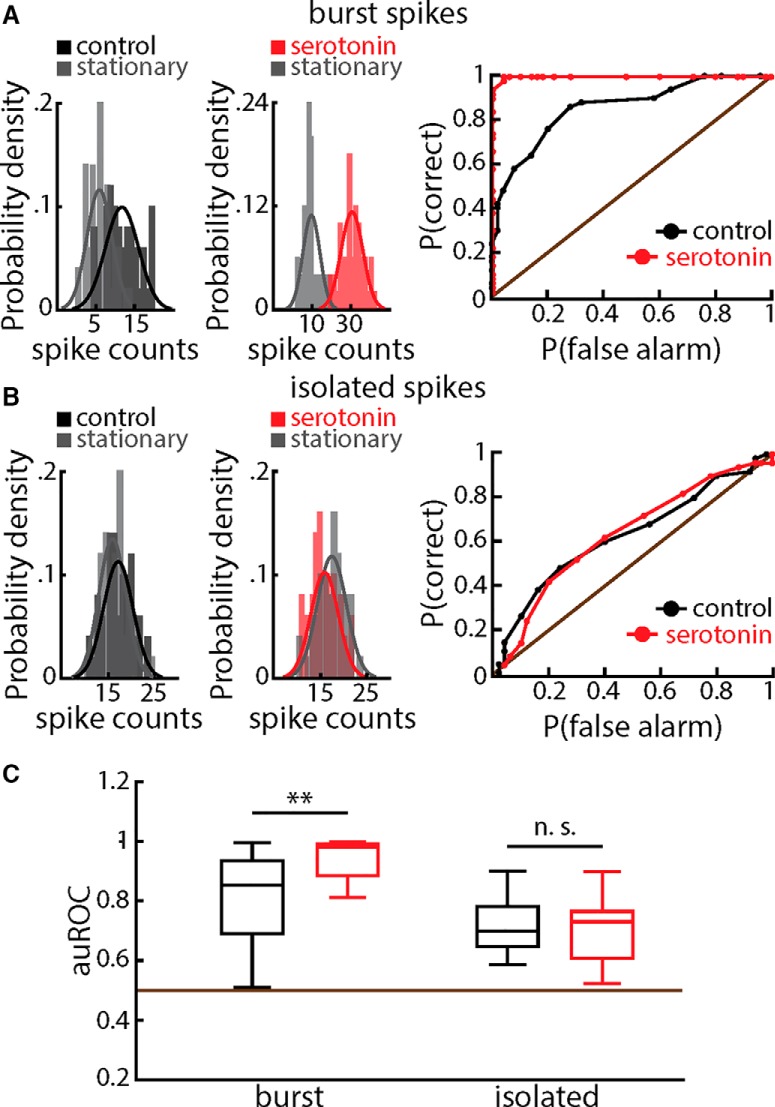
Serotonin increases receding motion stimulus detectability through enhanced burst firing. ***A***, Left, spike count distributions obtained during baseline (gray) and during receding stimulation (black) before serotonin application. Middle, spike count distributions obtained during baseline (gray) and during receding stimulation (red) after serotonin application. Right, ROC curves before (black) and after (red) serotonin application. We used the same example ELL pyramidal cell as in Figure 4. ***B***, Same as ***A*** but for isolated spikes. ***C***, Population-averaged auROC values for bursts (left) and isolated spikes (right) before (black) and after (red) serotonin application. A significant increase was observed for burst but not for isolated spikes (burst spikes: signed-rank test, *p* =.0134; isolated spikes: *t* test, *t*_(12)_ = 0.34, *p* = 0.737, *n* = 13).

Comparison of Figure 5*B*,*C* suggests that increased stimulus detectability during receding motion stimulation was due to a stronger increase in burst firing than during baseline. To test this hypothesis, we separated the spike trains during receding motion stimulation and during baseline into bursts and isolated spikes and then applied ROC analysis to both. Our results confirmed our hypothesis, as serotonin application significantly increased the discriminability of the burst spike count distributions (Fig. 6*A*, compare left and middle panels), as reflected in the ROC curves (Fig. 6*A*, right panel). In contrast, serotonin application did not affect the discriminability of the isolated spike count distributions (Fig. 6*B*, compare left and middle panels), as reflected in the ROC curves (Fig. 6*B*, right panel). Across our dataset, receding motion stimulus detectability as quantified by the auROC was significantly increased after serotonin application when considering bursts but not when considering isolated spikes (Fig. 6*C*).

### Serotonin application does not alter detectability when using a matching contrast paradigm

**Figure 7.**
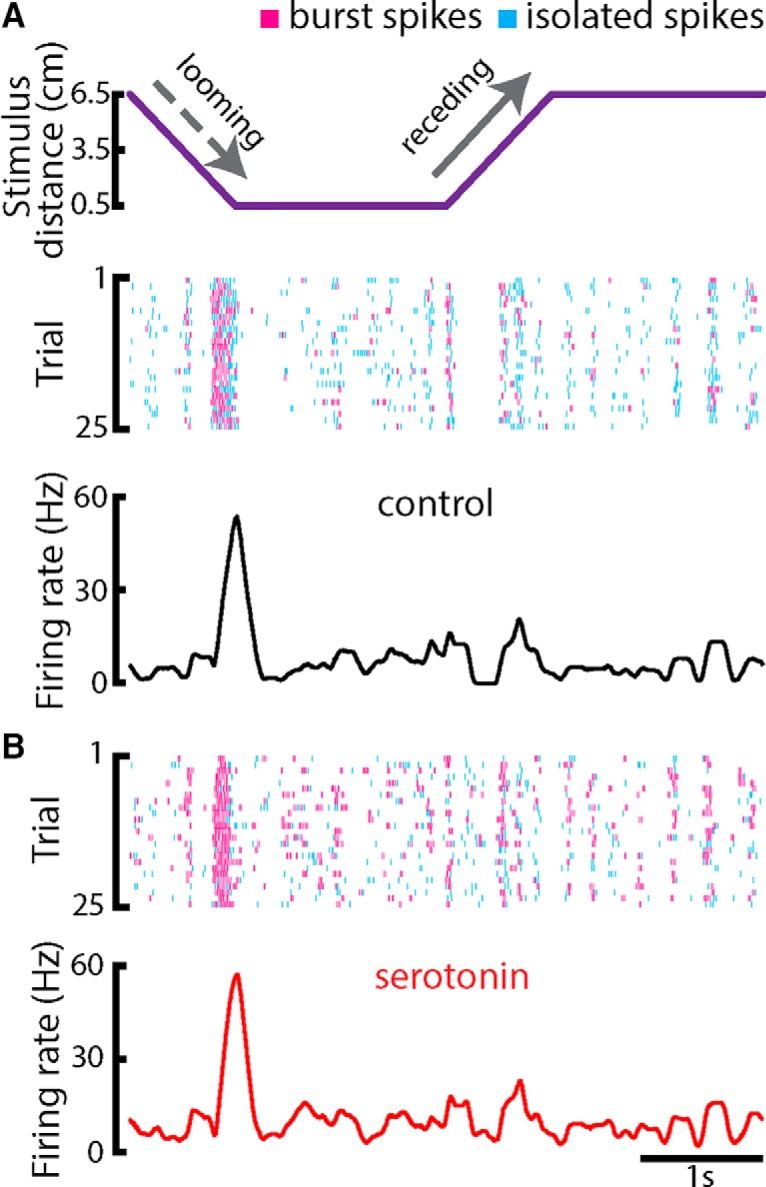
Serotonin enhances burst firing during a matching contrast paradigm. ***A***, Top, object position (lateral distance to the animal’s skin surface) as a function of time. The object’s speed during both looming and receding motion was 8 cm/s. Middle, Raster plot showing an example ELL pyramidal neuron’s spiking response to 25 stimulus presentations (i.e., trials) during control condition. The spikes that belong to bursts are shown in magenta whereas isolated spikes are shown in cyan. Bottom, PSTH of the neuron computed from 50 trials using all spikes. ***B***, Top, raster plot showing the example neuron’s response to 25 trials after serotonin application. Bottom, PSTH computed from 50 trials using all spikes.

**Figure 8.**
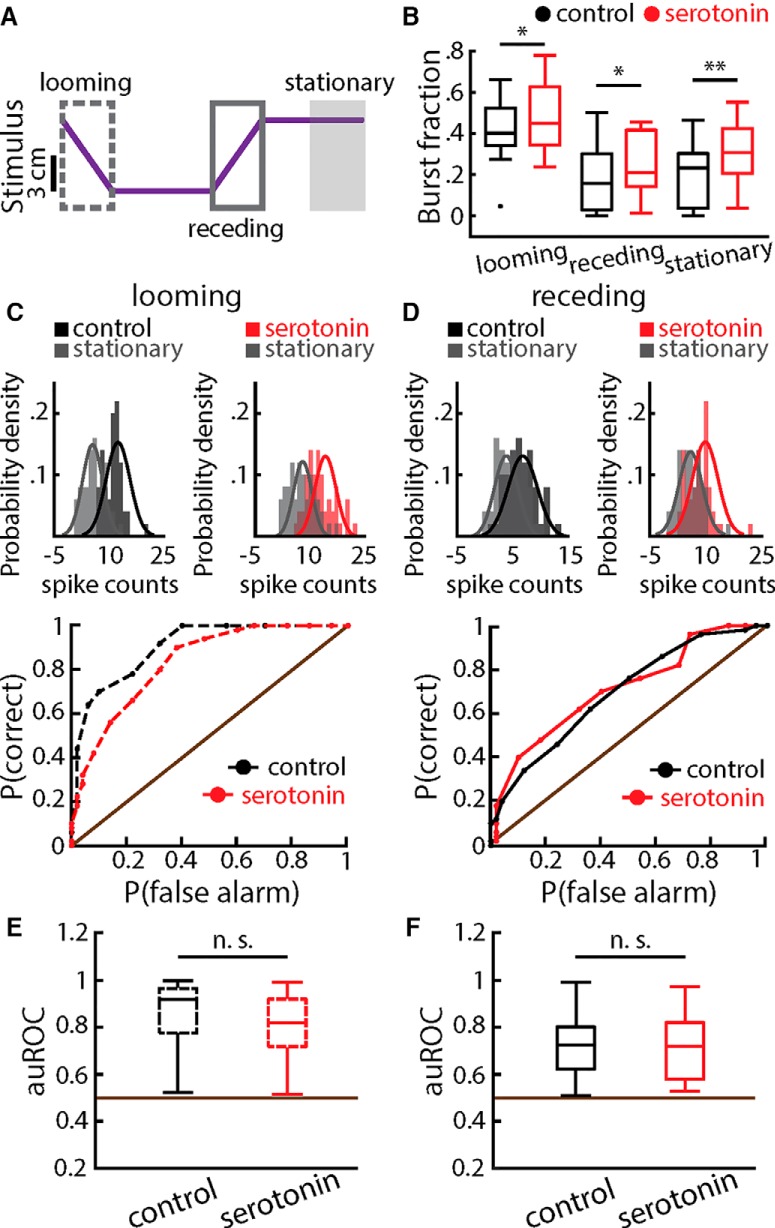
Serotonin does not alter stimulus detectability during at matching contrast paradigm. ***A***, Same as in Figure 4*A*, object position during a matching contrast paradigm. ***B***, Population-averaged burst fractions before (black) and after (red) serotonin application during looming (left), receding (middle), and baseline (right). Stars indicate statistical significance using a signed-rank test (looming: *p* = 0.0391, *n* = 9; receding: *p* = 0.0273, *n* = 9; baseline: *p* = 0.0039, *n* = 9). ***C***, Top, spike count distributions obtained during baseline (gray) and during looming stimulation (black or red) before (left) and after (right) serotonin application for an example ELL pyramidal cell. Best-fit Gaussian curves are superimposed on each distribution. Bottom, ROC curves from this example neuron before (black) and after (red) serotonin application. ***D***, Same as ***C*** but for receding motion. ***E***, Population-averaged auROC before (black) and after (red) serotonin application corresponding to looming motion. The horizontal brown line indicates the chance level. The observed decrease after serotonin application was not significant (signed-rank*, p* = 0.25, *n* = 9). ***F***, Same as ***E*** but for receding motion. No significant change was observed (signed-rank*, p* = 0.7344, *n* = 9).

We further tested the effects of serotonin on responses to motion using a matching contrast paradigm (i.e., ON cells were stimulated with a metal object while OFF cells were stimulated with a plastic object). We found that looming motion caused a strong increase in spiking activity (Fig. 7*A*,*B*, dashed gray arrow), while receding motion instead caused decreased spiking activity (Fig. 7*A*,*B*, solid gray arrow). Serotonin application significantly increased burst firing during all phases of motion (Fig. 8*A*,*B*), which is consistent with results obtained above using the non-matching contrast paradigm. However, we found that enhanced burst firing during either of looming or receding motion did not lead to enhanced detectability (Fig. 8*C–F*). This is most likely due to the fact that increases in burst firing during both looming and receding motion were similar to those observed during baseline activity (Fig. 8*B*).

### The effects of serotonin application are negatively correlated with the cell’s spontaneous firing rate

**Figure 9.**
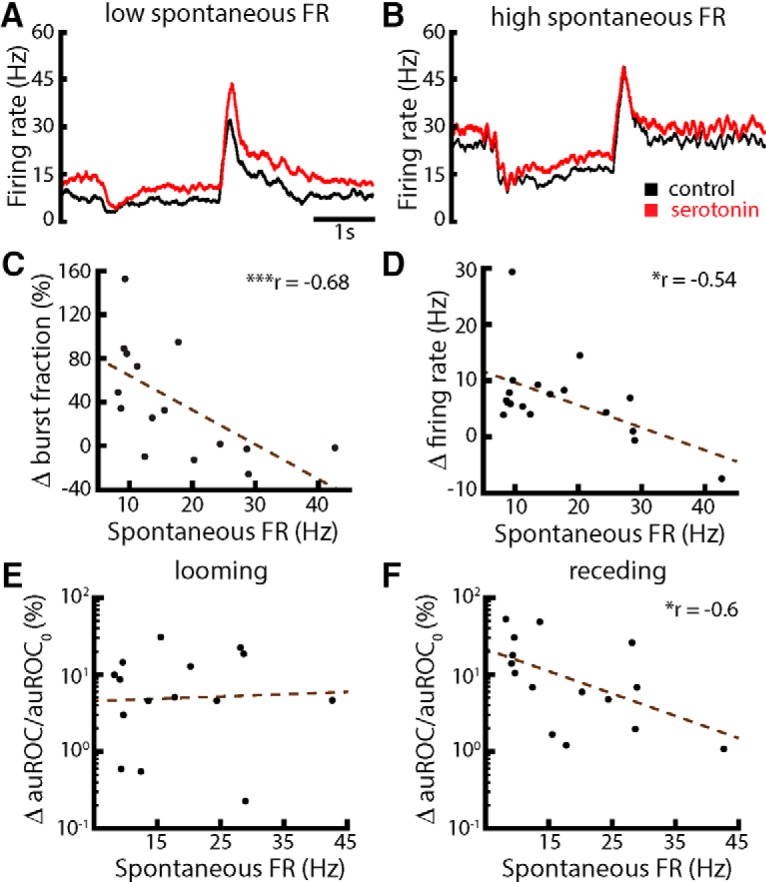
The magnitude of the effect of serotonin on ELL pyramidal cell’s responses to moving objects was negatively correlated with spontaneous firing rate. ***A***, PSTH of a representative cell with low (9.1 Hz) spontaneous firing rate during control (black) and after serotonin application (red). ***B***, Same as ***A*** but for a representative cell with high (28.9 Hz) spontaneous firing rate. For both panels, the time scale is the same and the object’s speed during looming and receding motion was 8 cm/s. ***C***, Percentage change in burst fraction [i.e., 100*(BF_serotonin_ – BF_control_)/BF_control_] between control and serotonin conditions (measured during baseline period) as a function of spontaneous firing rate measured before serotonin application. Both quantities were significantly correlated (*r* = −0.68, *p* = 0.003, *n* = 15). ***D***, Relative change in firing rate [i.e., 100*(FR_serotonin_ – FR_control_)/FR_control_] as a function of the spontaneous firing rate. Both quantities were significantly correlated (*r* = −0.54, *p* = 0.02, *n* = 15). ***E***, Percentage change in auROC [i.e., 100*(auROC_serotonin_ − auROC_control_)/auROC_control_] as a function of spontaneous firing rate for looming motion. No significant correlation was observed (*r* = −0.06, *p* = 0.84, *n* = 15). ***F***, Same as ***E*** but for receding motion. Both quantities were significantly correlated (*r* = −0.6, *p* = 0.03, *n* = 15).

We found that the effects of serotonin were heterogeneous as we observed strong effects for cells with lower spontaneous firing rates (Fig. 9*A*) and weaker effects for cells with higher spontaneous firing rates (Fig. 9*B*). Indeed, there was a strong negative correlation between the changes in burst fraction as well as firing rate due to serotonin application, and the cell’s spontaneous firing rate before serotonin application (Fig. 9*C*,*D*, respectively). Moreover, while there was no significant correlation between the relative change in detectability and the spontaneous firing rate for looming motion (Fig. 9*E*), we observed a significant correlation between both quantities for receding motion (Fig. 9*F*), when using a non-matching contrast paradigm. These results therefore confirm our hypothesis that the effect of serotonin is greatest in cells with low spontaneous firing rates and weakest in cells with high spontaneous firing rates.

### Effects of serotonin on ELL pyramidal cell responses to motion stimuli are speed invariant

**Figure 10.**
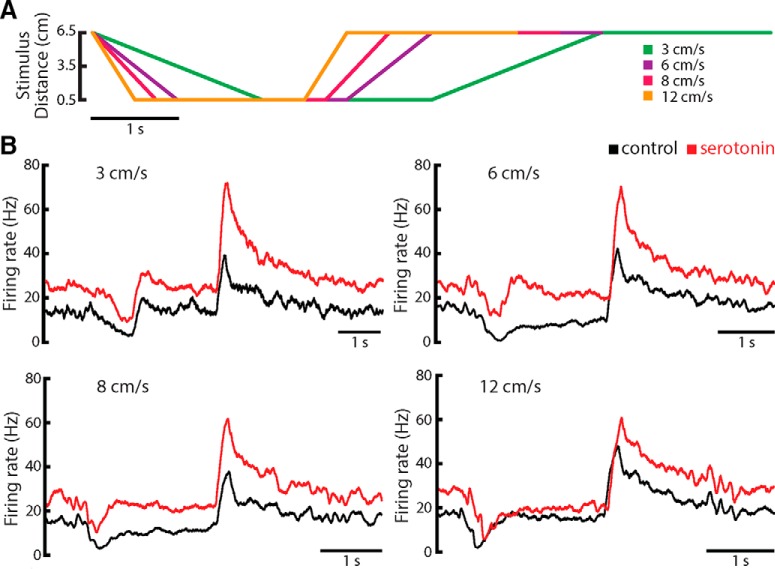
Serotonergic modulation of ELL pyramidal cell activity to motion is speed invariant. ***A***, Object position as a function of time. The object was moved at four different velocities: 3 cm/s (green), 6 cm/s (purple), 8 cm/s (pink), and 12 cm/s (orange). ***B***, PSTH of a representative neuron for each stimulus speed. All the curves follow the same shape with a strong increase in firing rate right after the onset of receding motion and inhibition right at the end of looming motion. Note that the firing rate is higher after serotonin application (red trace) than during control condition (black trace).

Finally, we investigated the effects of varying the speed of the moving object during both looming and receding motion when using a non-matching contrast paradigm. We tested velocities of 3, 6, 8, and 12 cm/s that are within the behaviorally relevant range (Bastian, 1982; Rose and Canfield, 1993a,b; Nelson and MacIver, 1999; Cowan and Fortune, 2007). The temporal profiles of the different stimuli are shown in Figure 10*A*. The responses of a typical ELL pyramidal cell before (black) and after (red) serotonin application are shown in Figure 10*B* for different object velocities. Overall, similar profiles were observed for each velocity in that the looming motion caused a decrease in firing rate that somewhat recovered while the object remained close to the animal. Receding motion furthermore caused a large increase in firing rate for all object velocities. The effects of serotonin were furthermore largely independent of object velocity in that there was an overall increase in firing rate and a large increase in the response to receding motion (Fig. 10*B*).

We then quantified the effects of serotonin on both looming and receding motion stimulus detectability using ROC analysis. Overall, we found that auROC values for looming motion stimuli before and after serotonin application were not significantly different from one another (3 cm/s: control: auROC = 0.69 ± 0.03; serotonin auROC = 0.71 ± 0.04; paired *t* test, *t*_(11)_ = −0.7010, *p* = 0.4979, *n* = 12; 6 cm/s: control: auROC = 0.65 ± 0.03; serotonin auROC = 0.68 ± 0.04; paired *t* test, *t*_(11)_ = −0.8437, *p* = 0.4153, *n* = 12; 8 cm/s: control: auROC = 0.63 ± 0.02; serotonin auROC = 0.60 ± 0.02; paired *t* test, *t*_(12)_ = 1.0812, *p* = 0.3009, *n* = 13; 12 cm/s: control: auROC = 0.64 ± 0.03; serotonin auROC = 0.63 ± 0.03; paired *t* test, *t*_(11)_ = 0.2727, *p* = 0.7902, *n* = 12). Overall, these values were not significantly different when comparing across the different speeds (one-way ANOVA, *F*_(3,12)_ *= 0.84*, *p* = 0.4768). In contrast, auROC values for receding motion stimuli significantly increased after serotonin application for all speeds (3 cm/s: control: auROC = 0.83 ± 0.04; serotonin auROC = 0.90 ± 0.04; paired *t* test, *t*_(11)_ = −2.4420, *p* = 0.0327, *n* = 12; 6 cm/s: control: auROC = 0.90 ± 0.04; serotonin auROC = 0.97 ± 0.01; signed-rank test, *p* = 0.0522, *n* = 12; 8 cm/s: control: auROC = 0.86 ± 0.03; serotonin auROC = 0.97 ± 0.01; signed-rank test, *p* = 0.0007, *n* = 12; 12 cm/s: control: auROC = 0.86 ± 0.04; serotonin auROC = 0.98 ± 0.01; signed-rank test, *p* = 0.0020, *n* = 12). However, these values were not significantly different when comparing across the different speeds (one-way ANOVA, *F*_(3,12)_ = 0.43, *p* = 0.7304).

## Discussion

### Summary of results

We investigated the effects of serotonin on processing of looming and motion stimuli by ELL pyramidal cells. Overall, focal exogenous serotonin application increased pyramidal excitability, thereby leading to increases in firing rate as well as the tendency to fire bursts of action potentials during both looming and receding motion stimulation, as well as during the absence of stimulation and baseline. When using a non-matching contrast paradigm, we found that serotonin application increased detectability of receding but not looming motion stimuli because increases in burst firing were more pronounced during receding motion stimulation than during looming stimulation or baseline. Instead, when using a matching contrast paradigm, we found that serotonin application did not alter detectability of either of receding or looming motion because increases in burst firing during receding motion stimulation were similar to those occurring during looming stimulation or baseline. The effects of serotonin were greatest for pyramidal cells with low spontaneous firing rates and weakest for cells with high spontaneous firing rates. Finally, we showed that the effects of serotonin on responses to both looming and receding motion stimuli were robust as they were independent of object speed.

### Physiologic mechanisms by which serotonin enhances responses to receding motion

What are the underlying mechanisms by which serotonin enhances burst firing? The great similarity between the brain anatomies of *A. albifrons* and *A. leptorhynchus*, electrophysiological results showing largely similar ELL pyramidal cell neural responses (Bastian, 1981; Krahe et al., 2008; Khosravi-Hashemi and Chacron, 2014; Martinez et al., 2016), together with our results showing that serotonin application has an effect on pyramidal cell excitability that is similar to that observed previously for *A. leptorhynchus* (Deemyad et al., 2013), all strongly suggest that the mode of action of serotonin is shared between both species. In *A. leptorhynchus*, serotonin enhances pyramidal cell excitability *in vitro* by inhibiting outward currents mediated by SK and M-type channels (Deemyad et al., 2011), an effect that is mediated by 5-HT2-like receptors (Larson et al., 2014). Both currents give rise to a pronounced afterhyperpolarization (AHP) following an action potential, which opposes burst firing (Toporikova and Chacron, 2009; Deemyad et al., 2011). Strong attenuation of the AHP by serotonin allows for increased burst firing, which is mediated by a somato-dendritic interaction (Lemon and Turner, 2000). Specifically, somatic action potentials backpropagate into the proximal apical dendrites where they trigger a wider dendritic spike that propagates back to the soma, leading to a depolarizing after potential (DAP). The DAP at the soma grows in size throughout the burst, which leads to a progressive depolarization and a shortening of the interspike interval throughout the burst. The burst then terminates with a characteristic doublet when the interspike interval becomes shorter than the dendritic refractory period (Noonan et al., 2003). Indeed, intracellular recordings performed *in vivo* in *A. leptorhynchus* have shown that serotonin application decreases the AHP after the action potential, thereby revealing the DAP (Deemyad et al., 2013). Although further studies are needed to confirm this, it is very likely that serotonin application increases ELL pyramidal cell excitability in *A. albifrons* by inhibiting currents mediated by SK and M-type channels.

How does inhibition of SK and M-type currents lead to selective enhancement of detectability of receding stimuli when using a non-matching contrast paradigm? In *A. leptorhynchus*, only the SK1 and SK2 channel subtypes are expressed in the ELL (Ellis et al., 2007b, 2008), with SK1 channels expressed on the dendrites of ON and OFF-type ELL pyramidal cells and SK2 channels expressed near the somata of ON-type cells only (for review, see Huang and Chacron, 2017). Since the effects of serotonin on ELL pyramidal cell excitability were similar for both ON- and OFF-type cells (Deemyad et al., 2013), it is thought that serotonin primarily inhibits SK1 channels, which is supported by anatomic results showing rich density of serotonergic fibers within the molecular layer (Deemyad et al., 2011) where pyramidal cell apical dendrites are located. Previous results obtained in *A. leptorhynchus* have shown that, when using a non-matching contrast paradigm, the increased firing rate response to receding motion was mediated by descending input from higher centers (Clarke and Maler, 2017). Instead, when using a matching contrast paradigm, Clarke and Maler (2017) have shown that excitatory responses were mediated primarily by ascending input from EA’s. ELL pyramidal cells receive large amounts of descending input that terminates within the molecular layer (Sas and Maler, 1983, 1987; Berman and Maler, 1999) and whose functions include gain control (Bastian, 1986a,b) as well as cancelation of self-generated and low frequency stimuli generated by conspecifics (Bastian, 1999; Bastian et al., 2004; Bol et al., 2011; Mejias et al., 2013). In contrast, ascending input from EA’s terminates on the basilar dendrites and somata of ELL pyramidal cells (Maler, 1979; Maler et al., 1981). As such, it is likely that the mechanism by which detectability of receding motion is enhanced by serotonin in *A. albifrons* is through inhibition of dendritic SK1 (as well as possibly M-type) channels, which suppresses the underlying AHP and enhances burst firing more effectively in response to descending rather than in response to ascending input. We hypothesize that this is because synapses from descending pathways are located closer to SK1 (and possible M-type) channels. If true, then this would explain our results showing that serotonin application selectively increased detectability during receding motion when using a non-matching contrast paradigm. Further studies are needed to verify these predictions.

We also note that previous studies in *A. leptorhynchus* have shown a strong correlation between pyramidal cell morphology and spontaneous firing rate (Bastian and Nguyenkim, 2001; Bastian et al., 2004). Indeed, cells with the lowest spontaneous firing rates tend to display the largest apical dendrites and receive the largest amount of feedback, while cells with the highest spontaneous firing rates instead tend to display small apical dendrites and receive weak if non-existent feedback (for review, see Maler, 2009). Interestingly, cells with the highest spontaneous firing rates tend to display weak SK channel expression, whereas cells with the lowest spontaneous firing rates tend to display the strongest SK channel expression (Ellis et al., 2007b, 2008; for review, see Huang and Chacron, 2017). As such, our results showing that serotonin had the strongest effect on cells with low spontaneous firing rates and minimal to no effect on the activity of cells with high spontaneous firing rates in *A. albifrons* is consistent with the hypothesis that serotonin downregulates SK1 channels, which are most expressed in cells with low spontaneous firing rates. Further studies are however needed to confirm this.

### Functional role of serotonergic pathways in the electrosensory system

What is the function of the serotonergic input to ELL pyramidal cells? One of the main effects of serotonin is to decrease aggressive behaviors across species (Fachinelli et al., 1989; Montoya et al., 2012) including weakly electric fish (Maler and Ellis, 1987; Deemyad et al., 2013). Importantly, serotonin levels are higher in more submissive individuals than in dominant ones (Larson and Summers, 2001). While it has been known for a long time that ELL pyramidal cells receive serotonergic input from the raphe nuclei (Johnston et al., 1990), the functional role of such input has remained largely unknown until recently. Deemyad et al. (2013) have shown that both exogenous and endogenous release of serotonin gave rise to increased responses to stimuli associated with same-sex conspecifics in *A. leptorhynchus* (Deemyad et al., 2013). Specifically, because of sex differences, females in this species tend to display lower EOD frequencies than males (Zakon et al., 2002). As such, the beat frequency (which is given by the difference in EOD frequency) tends to be lower during encounters between two males or two females than between a male and a female. Moreover, *A. leptorhynchus* also generate communication signals called “chirps” that consist of brief increases in EOD frequency that mainly consist of two types: Type I chirps that are elicited primarily during male-female interactions and Type II chirps that are elicited primarily during male-male interactions (Zakon et al., 2002). Interestingly, Deemyad et al. (2013) showed that serotonin application only enhanced ELL pyramidal neural responses to low frequency (<50 Hz) beats when these were spatially diffuse and Type II chirps. However, responses to spatially localized beat stimuli and to Type I chirps were unaffected. They therefore concluded that the function of the serotonergic pathway was to selectively enhance neural responses to stimuli associated with same-sex conspecifics in more submissive individuals. While the results of Deemyad et al. (2013) were obtained in *A. leptorhynchus*, the arguments made above strongly suggest that these will also apply to *A. albifrons*.

However, weakly electric fish also use their electrosense to detect objects in their environment. Behavioral studies have shown important looming and receding motion during prey capture behavior (Nelson and MacIver, 1999) as well as during exploration of novel objects (Graff et al., 2004; Hofmann et al., 2013). Our results showing that serotonin increases the detectability of receding motion stimuli suggests that serotonin will also affect responses to moving stimuli. The dense serotonergic innervation of ELL pyramidal cells within LS could thus be beneficial for determining the object’s location in 3D relative to that of the animal. The results of theoretical studies showing that cells with large RFs are beneficial for object localization in three dimensions (Brown and Bäcker, 2006), as observed for LS pyramidal cells (Shumway, 1989), supports our hypothesis. We propose that higher serotonin levels observed for more submissive individuals will also be beneficial for electrolocation in general. Such studies will require measuring serotonin levels in the ELL (e.g., using *in vivo* voltammetry) as done recently (Fotowat et al., 2016) of both submissive and dominant individuals and are beyond the scope of this paper. In general, our results suggest that the functional role of serotonergic pathways is to enhance ELL pyramidal neuron responses to descending input. As such, the detectability of a stimulus for which the response is at least partially mediated by feedback input should then be enhanced. Recent evidence suggests that responses to spatially diffuse Type II chirps (Marsat and Maler, 2012) as well as low frequency beats are mediated by descending input in *A. leptorhynchus* (Bastian et al., 2004; Chacron et al., 2005; Chacron, 2006). In contrast, both pharmacological inactivation and lesion of descending input have no significant effect on either high frequency beats or spatially localized stationary stimuli (Chacron et al., 2005; Chacron, 2006). We note that the effects of descending input on Type I chirps have not been investigated systematically to date. However, current experimental data supports our hypothesis that the function of the serotonergic system is to enhance ELL pyramidal cell responses to stimuli that elicit feedback input. Further studies should focus on how serotonin affects responses to other stimuli that have not been considered before, such as changes in the beat amplitude (i.e., the envelope) that occur during movement that have been the focus of recent studies (Huang and Chacron, 2016; Huang et al., 2016; Martinez et al., 2016; Zhang and Chacron, 2016).

### Implications for other sensory systems

Previous studies have shown strong similarities between the electrosensory system and the visual, auditory, and vestibular systems of mammals (Clarke et al., 2015a; Metzen et al., 2015). Together with the fact that the serotonergic system displays high conservation across vertebrate species (Parent, 1981), this suggests that our results will be applicable to other systems. In particular, as the ELL is a cerebellar-like structure, it is likely that our results will be applicable to other cerebellar-like structures such as the dorsal cochlear nucleus (DCN) that also receive large serotonergic innervation. A recent study is shown that serotonin application increases the excitability of DCN neurons via 5-HT2 receptors but had heterogeneous effects on neural responses to stimuli (Felix et al., 2017). We hypothesize that the function of the serotonergic input to DCN will also be to enhance neural responses to stimuli that elicit descending input. Recent evidence has shown that descending input, which is found ubiquitously in the central nervous system (Cajal, 1909; Holländer, 1970; Ostapoff et al., 1990; Sherman and Guillery, 2002), plays key roles in determining neural responses to sensory input (Manita et al., 2015; Kwon et al., 2016; Takahashi et al., 2016). Further studies are needed to understand how neuromodulators affect processing by modulating how sensory neurons integrate descending input.

## Synthesis

Reviewing Editor: Leonard Maler, University of Ottawa Decisions are customarily a result of the Reviewing Editor and the peer reviewers coming together and discussing their recommendations until a consensus is reached. When revisions are invited, a fact-based synthesis statement explaining their decision and outlining what is needed to prepare a revision will be listed below.

The authors have now done the extra experiments called for by the reviewers and editor. They have applied 5-HT during both looming and receding motion and for both matching and non-matching stimulus/cell type conditions.

The results now convincingly show that 5-HT selectively enhances the detectability of receding stimuli. This is an unexpected and interesting result.

I have only one request for a minor change that relates to the definition of ‘proximal’.

Please change:

“We hypothesize that this is because synapses from descending pathways are located more proximally to SK1...”

to

“We hypothesize that this is because synapses from descending pathways are located closer to SK1...”
